# Supplementary material for: Comparative Performance Evaluation of Continuous Monitoring Blood Culture Systems Using Simulated Septic Specimen
Source: Diagnostics (Basel). 2025 Feb 14;15(4):468. doi: 10.3390/diagnostics15040468 (PMC11854677; doi:10.3390/diagnostics15040468)
Supplement: Supplementary file 1 [file diagnostics-15-00468-s001.zip › diagnostics-3436364-supplementary.pdf]

Comparative Performance Evaluation of Continuous Monitoring Blood Culture Systems Using Simulated Septic Specimen

Kwangjin Ahn[1], Taesic Lee[2], Sangwon Hwang[3], Dong Min Seo[4], Young Uh[1*]

[1] Department of Laboratory Medicine, Yonsei University Wonju College of Medicine, Wonju-si, Republic of Korea

[2] Department of Family Medicine, Yonsei University Wonju College of Medicine, Wonju-si, Republic of Korea

[3] Department of Precision Medicine, Yonsei University Wonju College of Medicine, Wonju-si, Republic of Korea

[4] Department of Medical Information, Yonsei University Wonju College of Medicine, Wonju-si, Republic of Korea

[*]Corresponding author

Young Uh, M.D., Ph.D.

Department of Laboratory Medicine, Wonju Severance Christian Hospital, Yonsei University Wonju College of Medicine, 20 Ilsan-ro, Wonju 26426, Korea

E-mail: u931018@yonsei.ac.kr

Supplementary Data

Tables 3

Table S1. Test schedules 3

Table S2. Preparation of microorganism 4

Figures 5

Figure S1. Brochure for HubCentra84 for foreigner customers. 5

Figure S2. Exterior of HubCentra84. 6

Figure S3. Verification of colony-forming units of *Candida albicans*. 7

Figure S4. Verification of colony-forming units of *Staphylococcus aureus*. 8

Figure S5. Verification of the colony-forming unit of *Streptococcus pneumoniae.* 9

Figure S6. Verification of the colony-forming units of *Escherichia coli.* 10

Figure S7. Verification of colony-forming units of *Pseudomonas aeruginosa.* 11

Figure S8. Verification of colony-forming units of *Bacteroides fragilis.* 12

Tables

Table S1. Test schedules

Microorganism	Number of tests (date of test, year-month-day)		
	BacT/Alert® 3D	BACTEC™ FX	HubCentra84
Candida albicans	10 (2024-07-16)	-	10 (2024-07-16)
	10 (2024-08-01)	-	10 (2024-08-01)
	3 (2024-08-28)	10 (2024-08-28)	5 (2024-08-28)
	3 (2024-09-23)	10 (2024-09-23)	3 (2024-09-23)
	4 (2024-09-25)	10 (2024-09-25)	2 (2024-09-25)
Staphylococcus aureus	30 (2024-12-19)	30 (2024-12-19)	30 (2024-12-19)
Streptococcus pneumoniae	30 (2024-12-20)	30 (2024-12-20)	30 (2024-12-20)
Escherichia coli	10 (2024-07-23)	-	10 (2024-07-23)
	10 (2024-07-24)	-	10 (2024-07-24)
	5 (2024-09-10)	15 (2024-09-10)	5 (2024-09-10)
	5 (2024-09-11)	15 (2024-09-11)	5 (2024-09-11)
Pseudonomas aeruginosa	10 (2024-07-30)	-	10 (2024-07-30)
	10 (2024-07-31)	-	10 (2024-07-31)
	4 (2024-08-14)	10 (2024-08-14)	-
	3 (2024-08-20)	10 (2024-08-20)	-
	3 (2024-08-30)	10 (2024-08-30)	10 (2024-08-30)
Bacteroides fragilis	25 (2024-12-23)	25 (2024-12-23)	25 (2024-12-23)
	5 (2024-12-27)	5 (2024-12-27)	5 (2024-12-27)

Table S2. Preparation of microorganism

Microorganism (strain)	Dilution factor	Mean CFU/mL (IQR)	CFU of inoculation
Candida albicans (ATCC 14053)	$2x10^5$	4.4 (1–4)	13.2
Staphylococcus aureus (ATCC 25923)	$4x10^6$	5.2 (4–8)	15.6
Streptococcus pneumoniae (ATCC 49619)	$4x10^6$	3.6 (0–7)	10.8
Escherichia coli (ATCC 25922)	$4x10^6$	2.0 (0–4)	6.0
Pseudomonas aeruginosa (ATCC 27853)	$4x10^6$	2.0 (0–4)	6.0
Bacteroides fragilis (ATCC 25285)	$4x10^6$	9.6 (4–15)	28.8

Abbreviations: CFU, colony-forming unit; IQR, interquartile range; ATCC, American Type Cul-ture Collection.

Figures

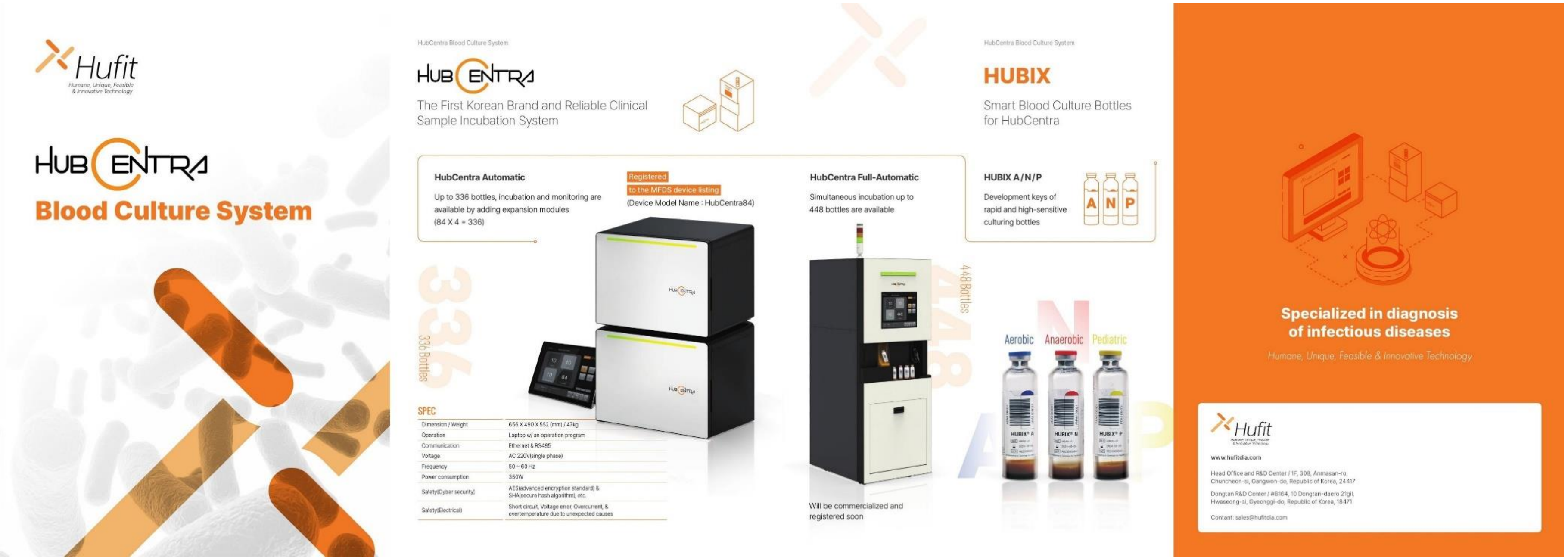

Figure S1. Brochure for HubCentra84 for foreigner customers.

The guide highlights both the basic specifications of HubCentra84 and other product with advanced features. Currently, the website (https://www.hufitdia.com/) is available only in Korean for domestic customers; however, plans are in place to launch an English version by January 2025, providing support for overseas customers.

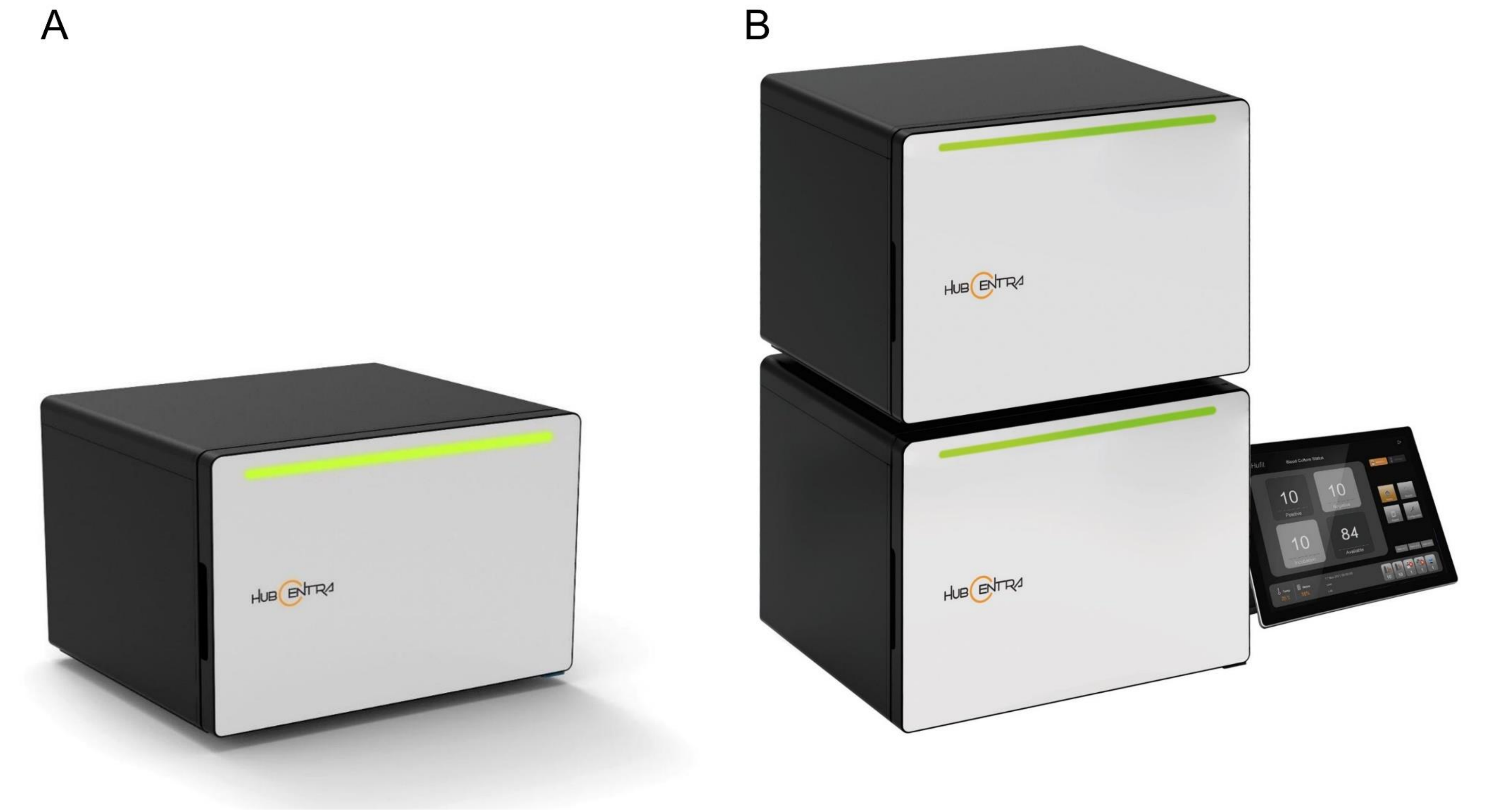

Figure S2. Exterior of HubCentra84.

(A) Appearance of a single unit. (B) Configuration showing two connected units in modular setup. Manageable via a portable laptop.

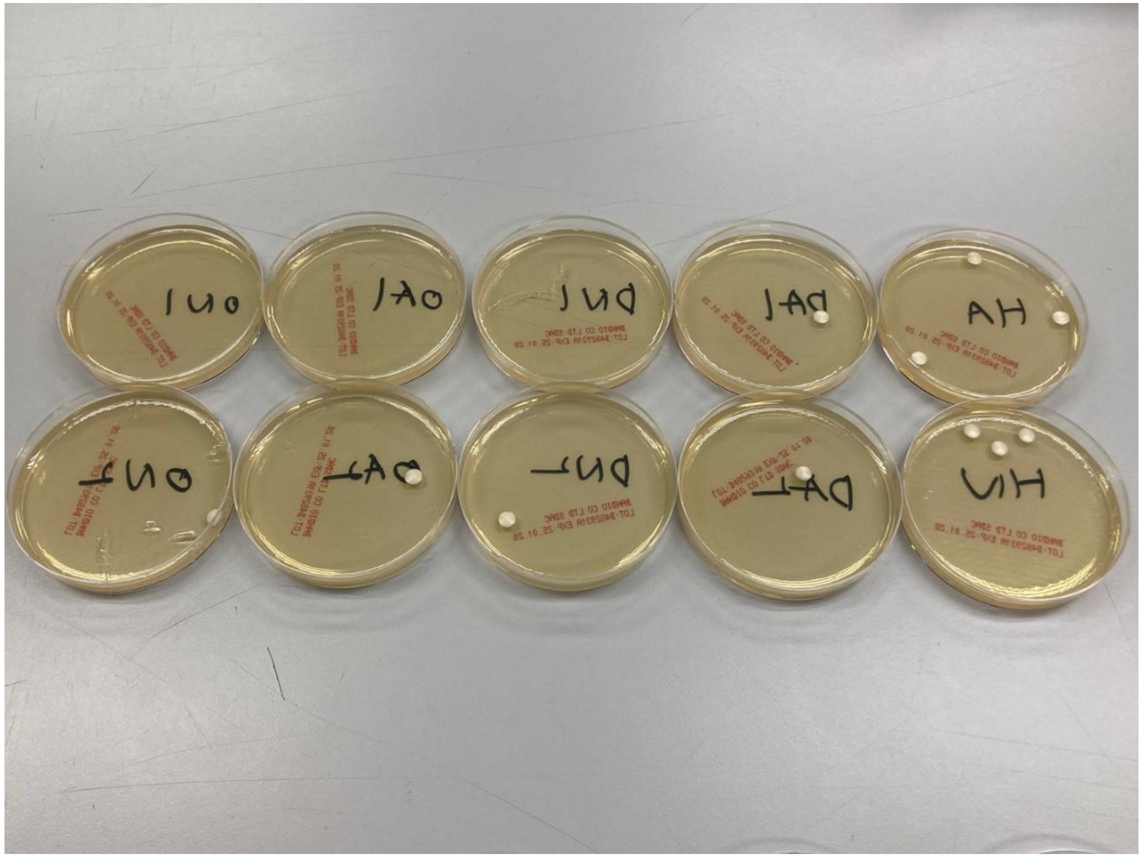

Figure S3. Verification of colony-forming units of *Candida albicans*.

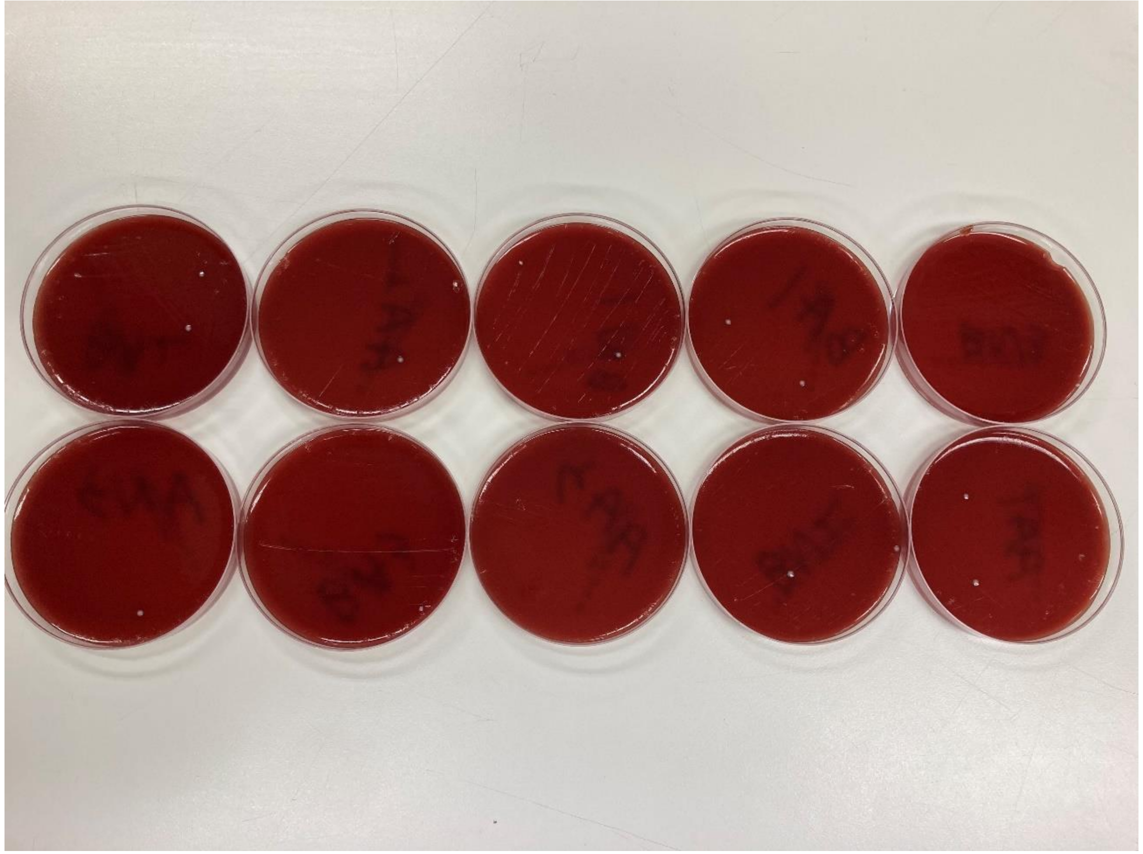

Figure S4. Verification of colony-forming units of *Staphylococcus aureus*.

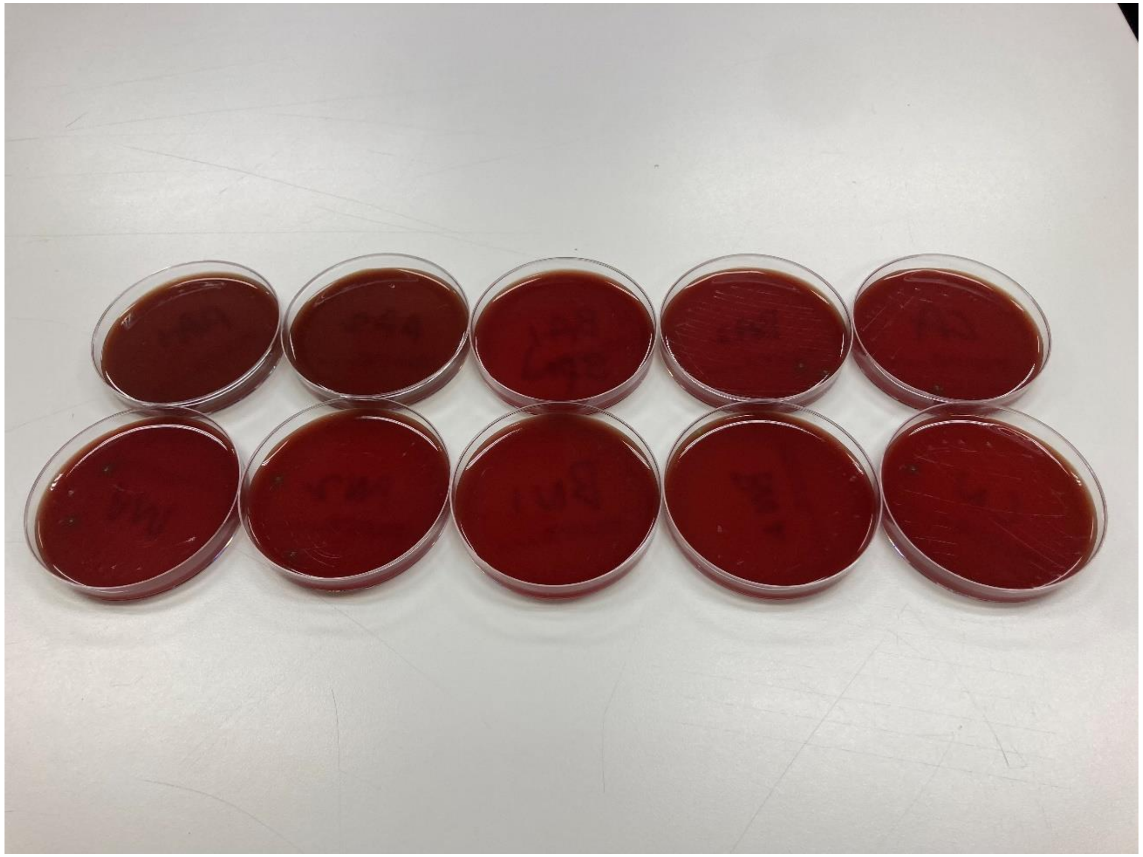

Figure S5. Verification of the colony-forming unit of *Streptococcus pneumoniae.*

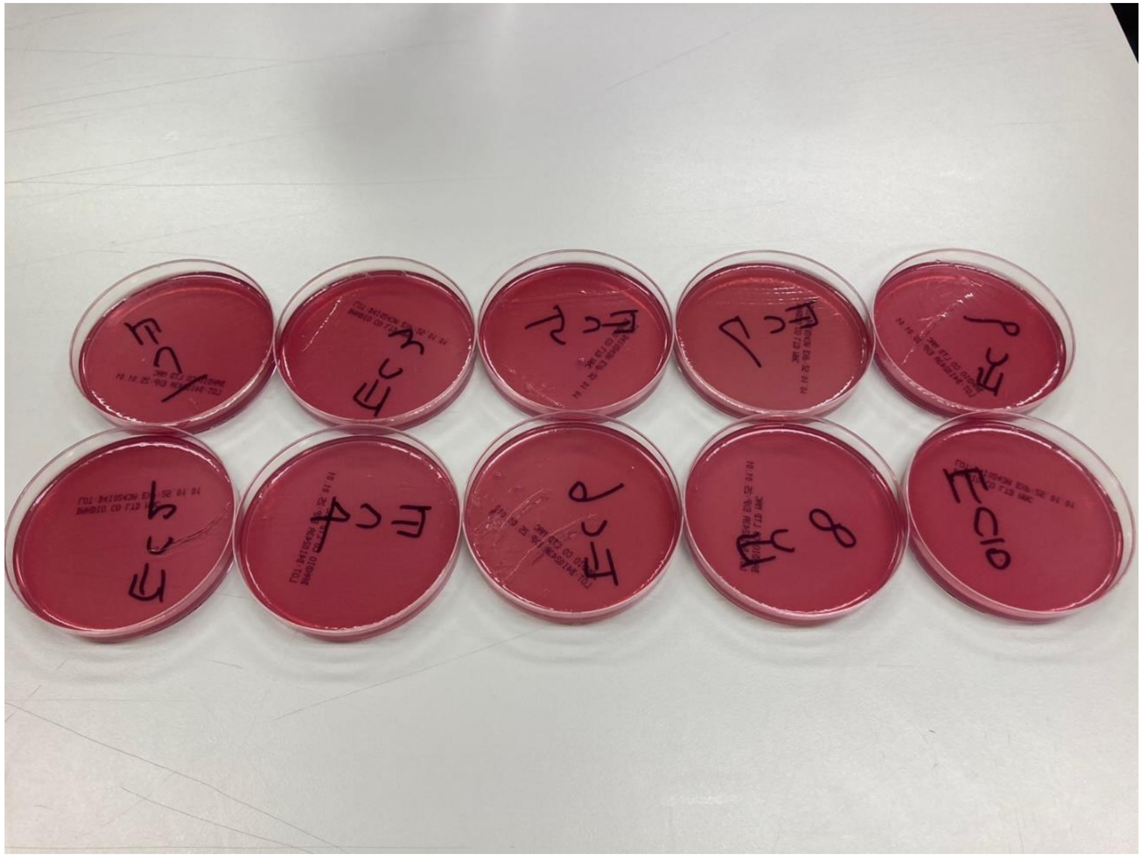

Figure S6. Verification of the colony-forming units of *Escherichia coli.*

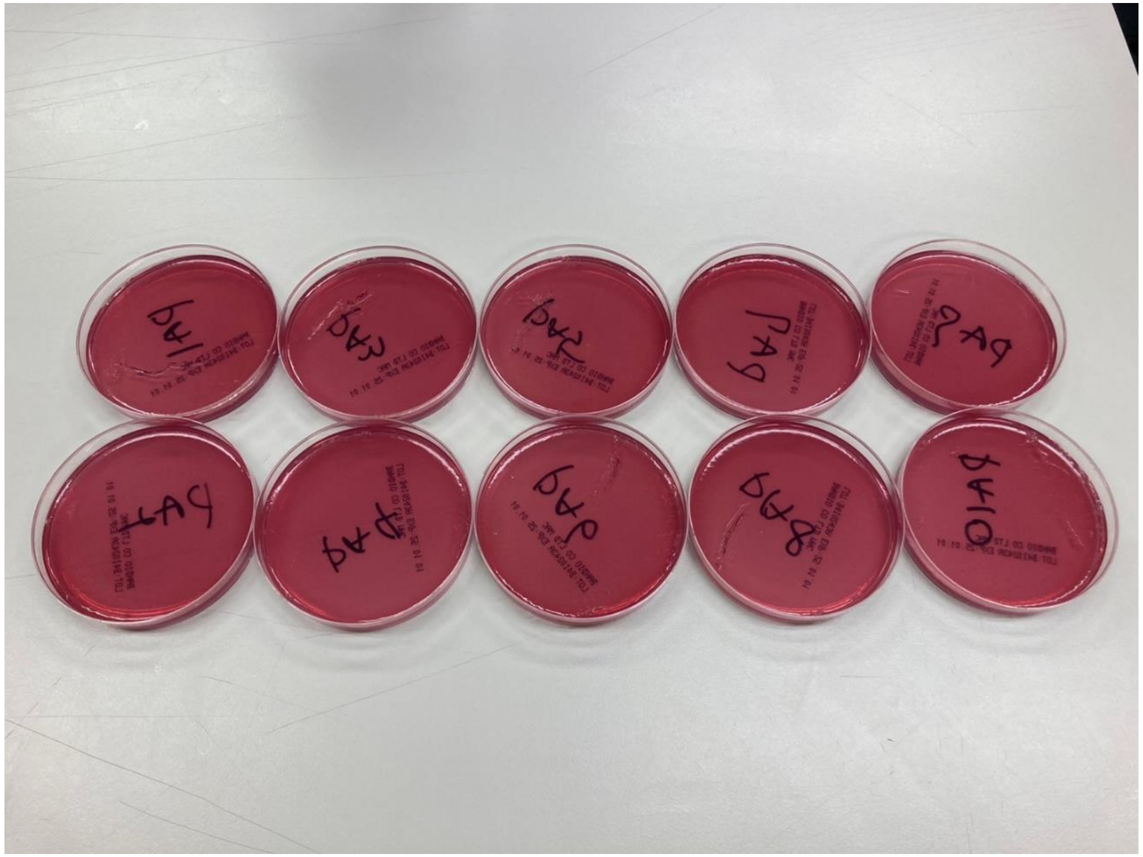

Figure S7. Verification of colony-forming units of *Pseudomonas aeruginosa*.

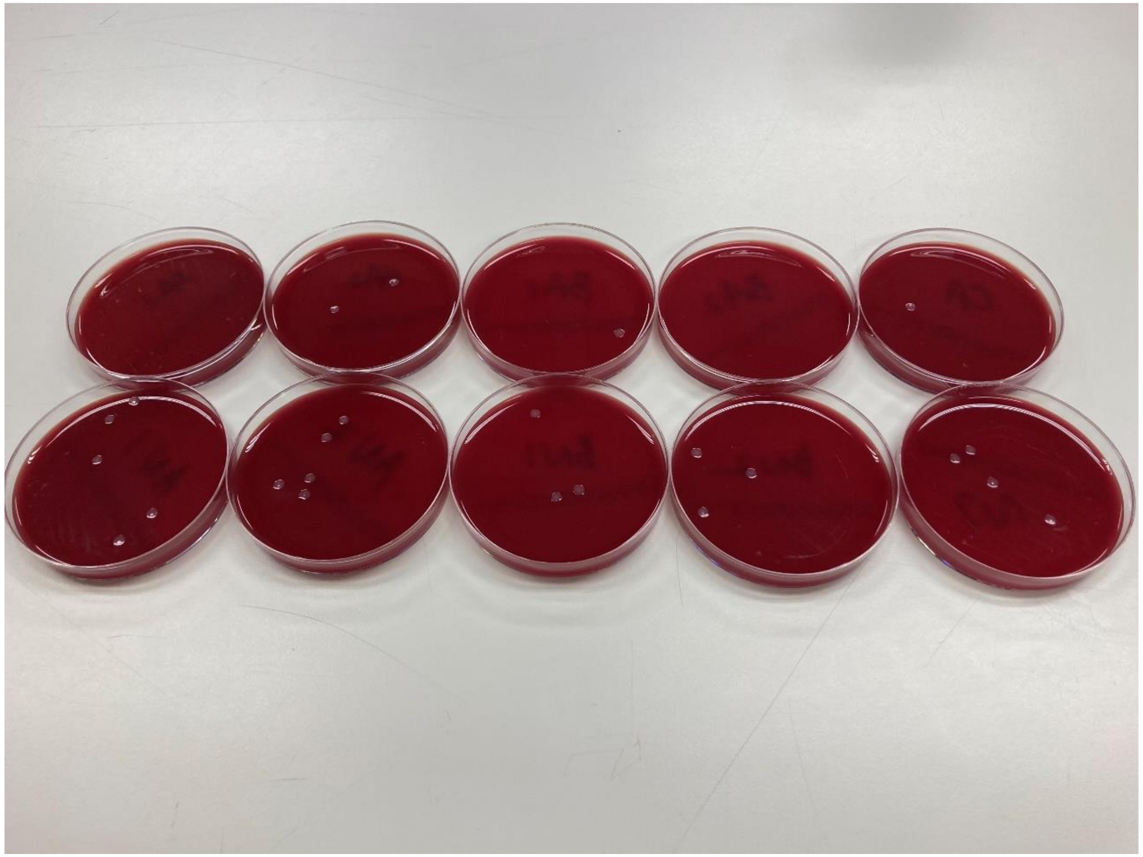

Figure S8. Verification of colony-forming units of *Bacteroides fragilis.*
